# Correlation Between Antimicrobial Consumption and Resistance in *Klebsiella pneumoniae* During the COVID-19 Pandemic Using Dynamic Regression Models: A Quasi-Experimental Epidemiological Time-Series Study

**DOI:** 10.3390/antibiotics14101020

**Published:** 2025-10-14

**Authors:** Paul Laffont-Lozes, Florian Salipante, Paul Loubet, Catherine Dunyach-Remy, Jean-Philippe Lavigne, Albert Sotto, Romaric Larcher

**Affiliations:** 1 Department of Pharmacy, Nimes University Hospital, 30029 Nimes, France; 2 Department of Biostatistics, Epidemiology, Public Health, and Innovation in Methodology (BESPIM), University of Montpellier, Nimes University Hospital, 30029 Nimes, France; 3 VBIC, INSERM U1047, University of Montpellier, Department of Infectious and Tropical Diseases, Nimes University Hospital, 30000 Nimes, France; 4 VBIC, INSERM U1047, University of Montpellier, Department of Microbiology and Hospital Hygiene, Nimes University Hospital, 30000 Nimes, France; 5 PhyMedExp, INSERM U1046, CNRS, University of Montpellier, Department of Infectious and Tropical Diseases, Nimes University Hospital, 30029 Nimes, France

**Keywords:** COVID-19, *Klebsiella pneumoniae*, dynamic regression, antimicrobial consumption, antimicrobial resistance

## Abstract

**Background/Objectives**: The COVID-19 pandemic has been reported to impact antimicrobial consumption (AMC) and antimicrobial resistance (AMR) worldwide. We aimed to assess this correlation in *Klebsiella pneumoniae* before and during the COVID-19 pandemic and to estimate the burden of each antibiotic. **Methods**: We collected data on AMC of penicillins and beta-lactamase inhibitors (PBIs), anti-pseudomonal activity penicillins and beta-lactamase inhibitors (AAPBIs), cephalosporins, carbapenems, fluoroquinolones, aminoglycosides, and AMR in *K. pneumoniae* strains. The correlation between AMC and AMR was studied using dynamic regression models. **Results**: Overall, AMC of AAPBIs and fourth-generation cephalosporin increased, while fluoroquinolone consumption and AMR in the 2862 *K. pneumoniae* strains analyzed decreased. However, during the first year of the pandemic, we reported an increase in AMC and AMR. We found that 46% to 48% of the increase in cephalosporin, AAPBI, and fluoroquinolone resistance was explained by increased cephalosporin and fluoroquinolone consumption, 55% of the increase in PBI resistance was explained by increased PBI, cephalosporin, and fluoroquinolone consumption, and 58% of the increase in aminoglycoside resistance was explained by increased aminoglycoside consumption. **Conclusions**: During the COVID-19 pandemic, the increase in AMR in *K. pneumoniae* was correlated with the increase in AMC of several antibiotics, mainly cephalosporins and especially fluoroquinolones.

## 1. Introduction

Bacterial antimicrobial resistance (AMR) is increasing worldwide and is becoming a major global health concern [1]. The inappropriate consumption of antibiotics has been widely pointed out as one of the most important of the multiple factors involved in the development of bacterial AMR [1]. Soon after the onset of the coronavirus disease 2019 (COVID-19) pandemic, the negative effect of increased antimicrobial consumption (AMC) on AMR related to poor practices in the management of patients infected with severe acute respiratory syndrome coronavirus 2 (SARS-CoV-2) was reported in different countries [2]. Among strategies developed to combat AMR, antimicrobial stewardship programs have proven to be among the most effective [3]. Although international guidelines have been published to improve the management of COVID-19 and limit antimicrobial consumption in 2022 [4], the emergence of AMR largely affected the ESKAPE group of bacteria in 2020 and 2021 [2] and, in particular, *Klebsiella pneumoniae*, in our hospital and in others [5]. As the SARS-CoV-2 continues to spread worldwide, there remains a need to further investigate the correlation between AMC and AMR during COVID-19 outbreaks and to determine which antibiotics should be prioritized as targets in antimicrobial stewardship programs.

Some studies explored the correlation between AMC and AMR during the pandemic [2], but few used time-series analysis, which is known to be one of the most reliable [6], particularly that based on dynamic regression (DR) models [7]. DR models are statistical methods used for analyzing time-series data where the relationship between variables changes over time [8]. Our team [7] and others [9] previously used this methodology to investigate the impact of AMC on the emergence of AMR, highlighting that a reduction in fluoroquinolone, cephalosporin, and carbapenem consumption explained up to 96% of AMR variation in several bacterial species, mainly Gram-negative bacilli. Thus, although data suggested an increase in resistance in Gram-negative bacilli in Europe during the COVID-19 pandemic [5,10], no study has demonstrated a statistical correlation between the increase in AMC and the emergence of AMR in *K. pneumoniae* using DR models in this period.

In the present study, we aimed to report trends in AMC and AMR in *K. pneumoniae* strains from a teaching hospital before and during the COVID-19 pandemic. We then assessed the correlation between AMC and AMR using DR models, and we estimated the fraction of AMR related to AMC and the burden of each antibiotic class during the first surges of the COVID-19 pandemic in France (2020–2021).

## 2. Results

### 2.1. Inpatient-Days

Between 2014 and 2019, the median number of inpatient-days per month was around 55,000 (IQR; 54,000–56,500). During this period, the number of hospitalized patients increased to around 60,000 inpatient-days per month. During the COVID-19 outbreak, the median number of inpatient-days was 54,000 per month (IQR; 53,500–56,500), with a maximum of 61,000 inpatient-days per month.

### 2.2. Trends in Antimicrobial Consumption Before and During the COVID-19 Pandemic

The trends in AMC before the COVID-19 pandemic are summarized in Table 1.

From 2014 to 2019, the AMC of broad-spectrum β-lactams, such as anti-pseudomonal activity penicillins and beta-lactamase inhibitors (AAPBI) (β = 1.43, *p* < 0.001) and fourth-generation cephalosporin (β = 0.127, *p* < 0.001), increased. In detail, their mean consumption per month increased from 9 to 15 defined daily dose (DDD)/1000 inpatient-days and from 0.5 to 1 DDD/1000 inpatient-days, respectively. The AMC of sulfonamide also increased during this period, from 3 to 5 DDD/1000 inpatient-days (β = 0.25, *p* = 0.006). On the contrary, we observed a significant decrease in fluoroquinolone consumption from 34 DDD/1000 inpatient-days to 18 DDD/1000 inpatient-days (β = −2.6, *p* < 0.001). Regarding the other antibiotic classes, AMC remained stable for carbapenems, cephalosporins, penicillins and beta-lactamase inhibitors (PBIs) and aminoglycosides (Table 1).

The trends in AMC before and during the COVID-19 pandemic are summarized in Table 2. During the COVID-19 pandemic, the AMC increased significantly, reaching a peak during spring 2020 (first COVID-19 surge in France). Particularly, the consumption of cephalosporins, sulfonamides, AAPBIs, carbapenems, and fourth-generation cephalosporins increased from 37 to 43 (+16%), 4 to 5 (25%), 15 to 20 (+33%), 9 to 13 (+44%), and 1 to 3 (+200%) DDD/1000 inpatient-days, respectively. However, the consumption of fluoroquinolones and PBIs decreased, and that of aminoglycosides remained stable.

### 2.3. Trends in Antimicrobial Resistance Before and During the COVID-19 Pandemic

The trends in AMR are presented in Table 3.

**Figure 1:**
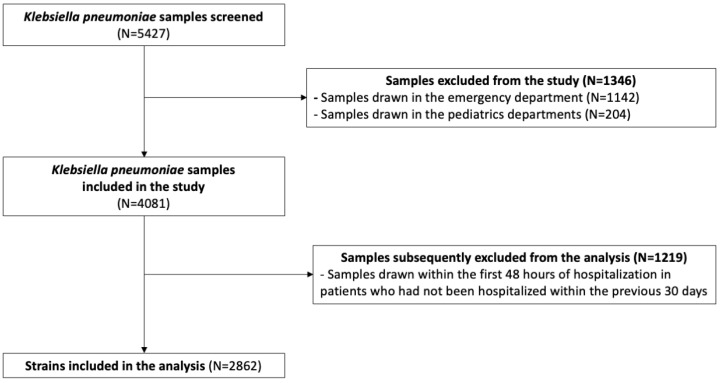
Flowchart of the study population.

Among the 4081 samples screened, 2862 samples were included in the analysis (Figure 1). Microbiological samples were mainly collected in rehabilitation units (*n* = 492, 17%), intensive care units (*n* = 424, 15%), surgical wards (*n* = 410, 14%), long-term care wards (*n* = 223, 8%), geriatric departments (*n* = 212, 7%), and gynecology departments (*n* = 134, 5%).

Over the study period, the rates of AMR to all antimicrobial classes decreased, with the exception of resistance to carbapenems, which remained stable (β = −0.08, *p* = 0.59). The decrease in AMR was particularly marked for fourth-generation cephalosporins, from 46% to 18% (β = −4.74, *p* < 0.001), and fluoroquinolones, from 46% to 25% (β = −3.67, *p* < 0.001).

**Figure 2:**
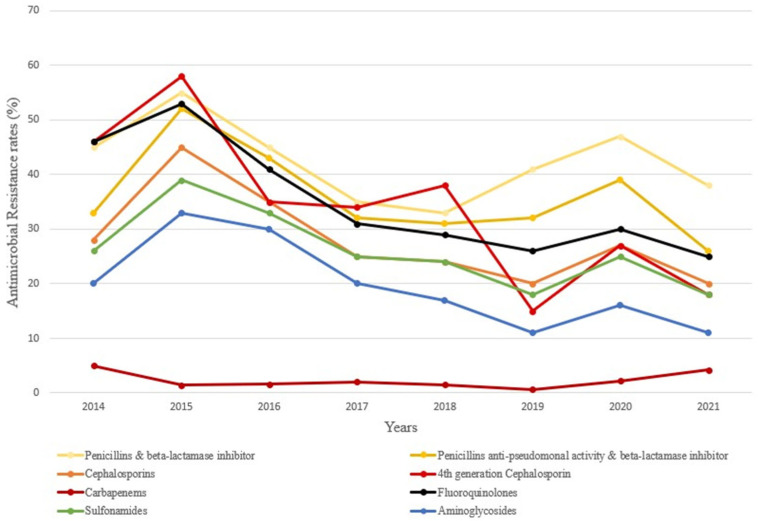
Annual antimicrobial resistance rates to the different classes of antibiotics in *Klebsiella pneumoniae* strains before (2014–2019) and during (2020–2021) the coronavirus disease 2019 (COVID-19) pandemic (AAPBIs: anti-pseudomonal activity penicillin and beta-lactamase inhibitors. PBIs: penicillin and beta-lactamase inhibitors).

**Figure A1:**
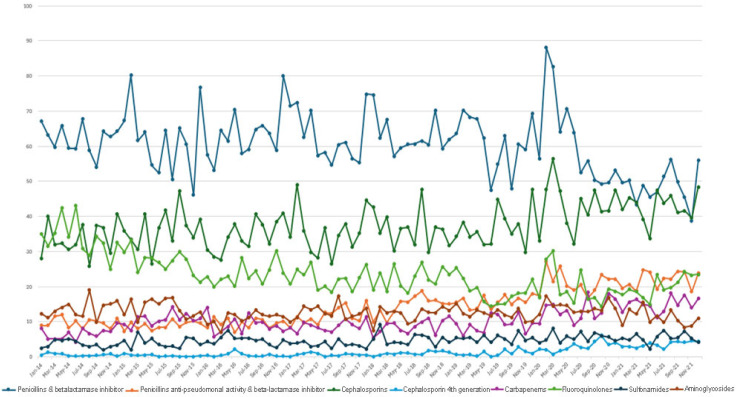
Monthly antimicrobial resistance rates to the different classes of antibiotics in *Klebsiella pneumoniae* strains before (2014–2019) and during (2020–2021) the coronavirus disease 2019 (COVID-19) pandemic.

However, during the COVID-19 pandemic, the AMR to all antimicrobial classes peaked in 2020 (Figure 2 and Figure A1). Specifically, between 2019 and 2020, we observed an increase in PBI, fluoroquinolone, AAPBI, cephalosporin, sulfonamide, aminoglycoside, fourth-generation cephalosporin, and carbapenem resistance rates of 15%, 16%, 22%, 35%, 39%, 45%, 80%, and 266%, respectively.

### 2.4. Correlation Between Antimicrobial Consumption and Antimicrobial Resistance During the COVID-19 Pandemic (2020–2021)

**Figure 3:**
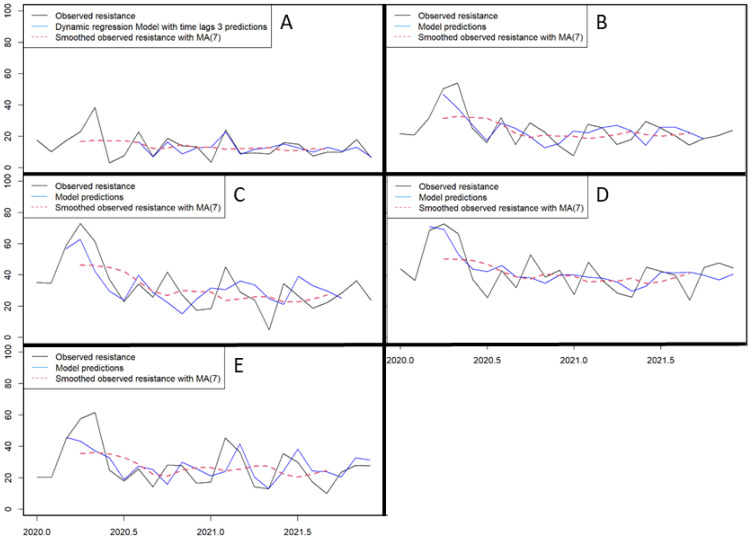
Dynamic regression modeling of (**A**) aminoglycosides resistance, (**B**) cephalosporins resistance, (**C**) penicillins anti-pseudomonal activity and beta-lactamase inhibitor resistance, (**D**) penicillins and beta-lactamase inhibitor resistance, and (**E**) fluoroquinolones resistance, in *Klebsiella pneumoniae*, during the coronavirus disease 2019 (COVID-19) pandemic (2020–2021). MA(7): moving average smoother with window size of 7 months; applied for graphical representation to highlight overall trends and reduce short-term fluctuations.

Using dynamic regression modeling (Figure 3), we found that AMR was mainly correlated with AMC, with a lag time of 1 to 3 months (Table 4 and Table 5). Resistance to cephalosporins was correlated with the AMC of three classes, namely cephalosporins, fourth-generation cephalosporins, and fluoroquinolones (Table 4). The change in AMC in these three classes of antimicrobials explained 47% of the changes in resistance to cephalosporins (Table 5). DR models showed that the AMC of the same three antibiotic classes explained 48% of the resistance to AAPBIs (Table 4 and Table 5), whereas PBI resistance was correlated with the consumption of PBIs, cephalosporins, and fluoroquinolones (Table 4), and 55% of resistance was explained by AMC in the DR model (Table 5). Resistance to fluoroquinolones was correlated with cephalosporin consumption (Table 4), whereas 46% of the fluoroquinolone resistance was explained by cephalosporin and fluoroquinolone consumption in the DR model (Table 5). Finally, resistance to aminoglycosides was only correlated with aminoglycoside consumption (Table 4). The aminoglycoside consumption explained 58% of the AMR to this class in the DR model (Table 5).

## 3. Discussion

We reported the results of a large retrospective epidemiological study, including nearly 3000 microbiological samples, that assessed the impact of AMC on AMR in *K. pneumonia* during the COVID-19 pandemic in a French tertiary hospital. Overall, AMR decreased over the study period (2014–2021); however, we observed a significant increase in 2020, during the first wave of the COVID-19 outbreak, which correlated with an increase in AMC. Using DR models, we highlighted that between 46% and 58% of the AMR emergence in *K. pneumonia* during the COVID-19 pandemic was explained by the consumption of several antibiotics, mostly cephalosporins and fluoroquinolones.

Before the COVID-19 pandemic, we observed that AMR was stable or decreasing in our hospital, consistent with data from other European countries [3]. Within this general trend, we noticed an increase in AMR in 2014–2015, which could have been favored by the opening of additional medico-surgical and hematology intensive care beds in our hospital that year. These units are known for being heavy consumers of antibiotics and contributors to AMR [7]. Accordingly, our study reported an increasing trend in the consumption of cephalosporin and carbapenem during this period. Our team has previously published data suggesting that certain environmental factors also contributed to the emergence of resistant *K. pneumoniae*, underlying the complexity of AMR emergence [11].

Most importantly and in agreement with previous studies [2,12], we reported an increase in AMC during the first wave of the pandemic. Some authors have suggested that antimicrobial overuse was related to the initial lack of awareness of the low rate of bacterial superinfections in COVID-19 [12]. Another contributing factor was the high rate of COVID-19 patients receiving mechanical ventilation [13], who, on the contrary, had a high rate of infection, especially ventilator-associated pneumonia [14]. In accordance, we found that the consumption of broad-spectrum antimicrobials, such as fourth-generation cephalosporins (+200%) and, to a lesser extent, carbapenems (+44%) increased the most, which has been highlighted in other European countries [15].

Our study highlighted that the strong impact of increased cephalosporin and fluoroquinolone consumption on the emergence of resistance to cephalosporins and fluoroquinolones, but also to AAPBIs and PBIs, in *K. pneumoniae* strains was one of the most interesting findings. These results are in agreement with studies using DR models in other settings, which have reported a correlation between cephalosporin resistance and the consumption of cephalosporins [16] and fluoroquinolones [17] and between fluoroquinolone resistance and consumption of fluoroquinolones and cephalosporins [18] in *K. pneumoniae*.

DR models present a valuable approach in studies dealing with the link between AMC and AMR, as they aim not only to assess potential correlations between AMC and AMR but also to quantify them. Our results revealed that the influence of AMC, mainly fluoroquinolones and cephalosporins, contributed to around half of the AMR emergence (46% to 58%), highlighting the burden of these antimicrobial classes relative to others in the study period. However, the DR models also highlighted that AMC accounted for only part of the emergence of AMR in *K. pneumoniae*, suggesting that physicians should also pay attention to other factors involved in the complex phenomenon of AMR, in particular the persistence of multidrug-resistant bacteria in the environment and transmission between patients [11].

In European countries, some authors reported similar findings [19,20]. However, we did not find a correlation between the increased consumption of carbapenems and the rate of carbapenem resistance. This could be explained by an increase in resistance from 0.6% to 2.2%, which involves only a few strains and, therefore, may not have been detected by our models. Another hypothesis could be the existence of an AMC threshold effect [21], which leads to the emergence of resistance, implying that our consumption, despite its increase, did not reach a level sufficient to generate selection pressure. The same was true for AABPIs, whereas both correlations have been widely reported elsewhere [16]. Our results also highlighted the weak impact of aminoglycoside consumption, which was only correlated with the emergence of aminoglycoside resistance, as previously reported by others [9].

Importantly, we previously showed that, from 2014 to 2019, the decrease in AMC was associated with a decrease in AMR in *Escherichia coli* and was driven by a decrease in fluoroquinolone consumption, while sulfonamide consumption increased [7]. Thus, our actual and previous findings [7] and those of others [3] strengthen the need for antimicrobial stewardship programs, limiting the consumption of fluoroquinolones. Strategies to restrict antibiotic misuse are mandatory to limit the emergence of multidrug-resistant *Enterobacterales*, such as extended-spectrum beta-lactamase and carbapenemase-producing *K. pneumoniae* [5], which have caused superinfections that have contributed to the increased mortality of SARS-CoV-2 infections [2,5].

The emergence of AMR is a complex and multifactorial phenomenon that is not solely linked to AMC in hospital settings. AMC in the community represent the largest amount of AMC (around 70% of the total AMC in France), and its burden on AMR emergence remains difficult to study [22]. Moreover, other events are involved in the emergence of AMR, such as colonization pressure that plays an important role in care structures [11]. During the COVID-19 pandemic, the infection prevention and control team was reinforced, and additional practices were implemented or strengthened, including systematic use of personal protective equipment (PPE), reinforced hand hygiene campaigns, stricter isolation procedures, and enhanced environmental cleaning and disinfection protocols, which may have influenced AMR through changes in colonization pressure. Additionally, the COVID-19 pandemic has raised concerns about its impact on AMR, with changes in antibiotic prescribing potentially contributing to inappropriate or excessive use, thereby worsening the issue [2,12]. Understanding these complex interactions is critical for developing effective strategies for managing and preventing AMR.

This study has several limitations. First, its single-center retrospective design may restrict the generalizability of the findings. Nonetheless, the inclusion of a large number of microbiological samples enhances the robustness of the analysis. DR models are accurate statistical tools for assessing the correlation between AMC and AMR due to their use of multiparametric input data [23]. Thus, while this study did not establish direct causality between AMC and AMR, it provides informative data to help physicians understand this complex phenomenon more deeply [20]. Moreover, several studies [8] corroborate our findings. Second, the nonlinear association between AMC and AMR may have prevented the identification of some correlations at low consumption and low resistance rates (threshold effect) [21]. Third, because EUCAST breakpoints and category definitions evolved during 2014–2021, we applied contemporaneous criteria year-by-year and performed sensitivity analyses. In our dataset, reclassification attributable to these updates affected ~1–3% of isolates overall and did not alter the direction of long-term trends. Fourth, the DR models used in this study did not incorporate the key mechanisms of AMR acquisition, such as colonization pressure and out-hospital AMC, nor individual factors like co-morbidities and antimicrobial dose In particular, although individual dose adjustments (e.g., in renal/hepatic dysfunction or intensive care) are not captured by DDDs, such variability is unlikely to alter hospital-level trends and is accounted for within the residual error of the regression models [23]. Moreover, focusing solely on hospital strains of *K. pneumoniae* limited their ability to provide a comprehensive understanding of AMR emergence, reducing their overall informational value, especially concerning its evolution in the community [1,11]. This limitation is further compounded by the exclusion of emergency department samples (including 47 taken from previously hospitalized patients, 1.6% of the population).

## 4. Materials and Methods

### 4.1. Study Design and Setting

We conducted a single-center retrospective epidemiological study at Nimes University Hospital using data from January 2014 to December 2021. This French tertiary hospital has 1786 beds, including 235 surgery beds, 46 intensive care unit beds, 24 haemato-oncology beds, and 190 long-term care beds.

### 4.2. Patients

During the study period, the number of annual admissions increased each year, from 112,000 to 144,000 patients per year admitted to the hospital. We searched the dates of hospital admission and discharge using medical software (Clinicom^®^, version 3.1, InterSystems Corporation, Cambridge, MA, USA) and calculated hospital length of stay.

### 4.3. Antimicrobial Consumption

Monthly data on AMC were extracted from pharmacy software (Pharma^®^, vesion 5.0, Computer Engineering, Paris, France). The AMC was determined by calculating the number of antimicrobials dispensed in care units and expressed in DDD per 1000 inpatient-days, following the ATC/DDD classification [23]. We focused on antibiotics commonly used to treat *K. pneumoniae* infections such as combinations of PBI (ampicillin-sulbactam and mainly amoxicillin-clavulanic acid), AAPBIs (namely, piperacillin-tazobactam and ticarcillin-clavulanic acid), third-generation cephalosporins (cefotaxime and ceftriaxone), fourth-generation cephalosporins (cefepime), carbapenems (ertapenem, imipenem, and meropenem), fluoroquinolones (norfloxacin, ofloxacin, ciprofloxacin, levofloxacin, moxifloxacin), aminoglycosides (gentamicin, tobramycin, amikacin), and sulfonamides (sulfamethoxazole–trimethoprim). The AMC data from the emergency department and the pediatrics department were not considered. Additionally, antimicrobials dispensed to outpatients were excluded from the analysis.

### 4.4. Bacterial Samples

We extracted microbiological data from the laboratory information system software (GLIMS, version 10, Clinisys Laboratory Solutions™, Chertsey, UK). We included in our analysis all microbiological samples (blood cultures, urine cultures, sputum, tracheal aspirates, bronchoalveolar lavage, cerebrospinal samples, peritoneal samples, or joint fluids or other diagnostic samples) positive for *K. pneumoniae* taken from inpatients during the study period. Surveillance samples like rectal swabs were not included. Microbiological samples obtained from outpatients, collected in the pediatrics or emergency departments, or within 48 h of hospital admission, were excluded from the analysis. However, some samples collected within 48 h of hospital admission were included if the patients had been previously hospitalized within the last 30 days, in accordance with the definition of nosocomial infection [24]. We deduplicated microbiological samples by considering patient identity and *K. pneumoniae* antibiotic susceptibility profiles, specifically focusing on phenotypic characterization (*K. pneumoniae* strains identified during the same sampling period are considered unique if their resistance profiles are identical). This process ensured that each unique combination of patient and antibiotic resistance profile was represented only once in the analysis.

The Department of Microbiology conducted bacterial identification using mass spectrometry Vitek^®^ MS (bioMérieux, Marcy-l’Etoile, France). Microbial samples are prepared and ionized using the MALDI method; then, the obtained mass spectra are compared to a reference library for identification. Antimicrobial susceptibility testing (AST) was performed using the Vitek 2^®^ automated system (AST-N372 card for urinalysis and AST-N233 card for other samples, bioMérieux) and/or the disk diffusion method on Mueller–Hinton agar (Bio-Rad, Hercules, CA, USA) according to the European Committee on Antimicrobial Susceptibility Testing guidelines. Resistance categorization and classes: AST results were interpreted using the EUCAST version in force in each calendar year. For class-level outcomes, an isolate was considered resistant to a class if it was non-susceptible to ≥1 agent in that class. Monthly resistance rates were computed as the non-susceptible percentage among tested isolates.

Finally, all isolates classified by resistant and intermediate categories were categorized as resistant. The resistance rate to a specific class of antimicrobials was calculated as the percentage of isolates resistant to at least one antimicrobial within that class out of the total isolates tested. This resistance rate was analyzed on a monthly basis for each class of antimicrobial.

### 4.5. Statistical Analysis

All statistical analyses were performed with R software version 4.2.0 (The R Foundation for Statistical Computing, Vienna, Austria) at 5% level of significance. Time-series analysis was conducted using the Forecast package [25]. Trends in AMC were assessed via linear regression, while Cochran–Armitage tests were used for AMR over the period 2014–2019. The slope of the linear trend AMR was expressed with the β coefficient. ARIMA models were utilized to depict *K. pneumoniae* resistance to various antibiotics. The ARIMA model was designed for analyzing and forecasting time-series data based solely on their past values and error terms; then, DR models were employed to investigate the relationship between the usage of multiple antimicrobials and resistance to the chosen antimicrobial during the COVID-19 pandemic (2020–2021). Unlike ARIMA, DR models extend this approach by incorporating external explanatory variables, thereby quantifying how changes in antimicrobial consumption (AMC) may influence antimicrobial resistance (AMR), while still accounting for autocorrelation and seasonality. Linear transfer function (LTF) was applied to link the output series (AMR) to the input series (AMC), considering potential lag time. To mitigate multicollinearity, a dimensionality reduction strategy, specifically dynamic regression by principal component (DRPC) models, was used, following the approach described by del Moral and Valderrama [26].

This model enables the consideration of multiple antibiotics at various lag times as input (AMC) to predict the output (AMR to a particular antibiotic). The model was initially computed using the first two principal components, then a Wald test was conducted, and only components with significant *p*-values were retained in the final model. Lag structure was determined empirically. For each antibiotic, we computed the cross-correlation function (CCF) between AMC and AMR after pre-whitening both series with their respective ARIMA models. Positive lags (AMC leading AMR) with CCF exceeding the 95% confidence limits were taken as candidate lags. Where multiple candidates existed, we selected the shortest clinically plausible lag and/or the lag yielding the best model fit (AIC) with adequate residual diagnostics (no remaining autocorrelation). The selected lags were then implemented in the linear transfer function within the dynamic regression. Robustness was checked by refitting models with ±1–2 time-step alternative lags. The coefficient of determination (R^2^) is reported to evaluate the percentage of the variance in the observed time-series explained by the model.

## 5. Conclusions

We observed a correlation between the increase in both AMC and AMR in *K. pneumoniae* during the COVID-19 pandemic in a French teaching hospital. Cephalosporins and fluoroquinolones were the main contributors to resistance. Over 2014–2021, reduced fluoroquinolone consumption correlated with decreased AMR. Our findings suggest antimicrobial stewardship should limit cephalosporin and fluoroquinolone consumption while favoring aminoglycosides and sulfonamides, which had a limited impact on AMR. Further studies are needed to confirm these results and evaluate the impact of stewardship programs on AMR and patient outcomes.

## Figures and Tables

**Table 1:** Trends in antimicrobial consumption (AMC) from 2014 to 2019.

Antimicrobial Class	AMC						Trends	
	2014	2015	2016	2017	2018	2019	2014–2019	*p*-Value
Aminoglycosides	14	14	11	13	12	12	No	0.08
Carbapenems	7	11	9	9	9	9	No	0.12
Fourth-generation cephalosporin	0.5	0.3	0.5	0.6	1	1	**Increase**	<0.001 *
Cephalosporins	34	36	34	35	36	37	No	0.11
PBI	62	62	63	63	63	62	No	0.74
AAPBI	9	9	18	11	14	15	**Increase**	<0.001 *
Fluoroquinolones	34	27	23	22	22	18	**Decrease**	<0.001 *
Sulfonamides	3	4	5	3	5	2	**Increase**	0.006 *

Annual AMC are expressed in mean defined daily doses/1000 inpatient-days. Aminoglycosides: amikacin, gentamicin, tobramycin; Carbapenems: meropenem, imipenem, ertapenem; Fourth-generation cephalosporin: cefepime; Cephalosporins: ceftriaxone, cefotaxime, ceftazidime; Penicillins and beta-lactamase inhibitors (PBI): amoxicillin-clavulanic acid, ampicillin-sulbactam; Anti-pseudomonal activity penicillins and beta-lactamase inhibitors (AAPBI): piperacillin-tazobactam, ticarcillin-clavulanic acid; Fluoroquinolones: ofloxacin, ciprofloxacin, levofloxacin, moxifloxacin; Sulfonamides: sulfamethoxazole-trimethoprim. Trend changed in bold. * significant *p*-value.

**Table 2:** Trends in antimicrobial consumption (AMC) from 2014 to 2021.

Antimicrobial Class	AMC								Trends	
	2014	2015	2016	2017	2018	2019	2020	2021	2014–2021	*p*-Value
Aminoglycosides	14	14	11	13	12	12	14	11	No	0.12
Carbapenems	7	11	9	9	9	9	13	15	**Increase**	<0.001 *
Fourth-generation cephalosporin	0.5	0.3	0.5	0.6	1	1	3	3	**Increase**	<0.001 *
Cephalosporins	34	36	34	35	36	37	43	43	**Increase**	<0.001 *
PBI	62	62	63	63	63	62	61	48	**Decrease**	0.002 *
AAPBI	9	9	18	11	14	15	20	22	**Increase**	<0.001 *
Fluoroquinolones	34	27	23	22	22	18	20	20	**Decrease**	<0.001 *
Sulfonamides	3	4	5	3	5	2	6	5	**Increase**	0.006 *

Annual AMC are expressed in mean defined daily doses/1000 inpatient-days. Aminoglycosides: amikacin, gentamicin, tobramycin; Carbapenems: meropenem, imipenem, ertapenem; Fourth-generation cephalosporin: cefepime; Cephalosporins: ceftriaxone, cefotaxime, ceftazidime; Penicillins and beta-lactamase inhibitors (PBI): amoxicillin-clavulanic acid, ampicillin-sulbactam; Anti-pseudomonal activity penicillins and beta-lactamase inhibitors (AAPBI): piperacillin-tazobactam, ticarcillin-clavulanic acid; Fluoroquinolones: ofloxacin, ciprofloxacin, levofloxacin, moxifloxacin; Sulfonamides: sulfamethoxazole-trimethoprim. Trend changed in bold. * significant *p*-value.

**Table 3:** Trends in antimicrobial resistance (AMR) from 2014 to 2021.

Antimicrobial Class	Resistance Rate in *Klebsiella pneumoniae* Strains (%)								Trends	*p*-Value *
	2014	2015	2016	2017	2018	2019	2020	2021		
Aminoglycosides	20	33	30	20	17	11	**16**	11	**Decrease**	<0.001 *
Carbapenems	5	1.4	1.6	2	1.5	0.6	**2.2**	4.2	No	0.242
Fourth-generation cephalosporin	46	58	35	34	38	15	**27**	18	**Decrease**	<0.001 *
Cephalosporins	28	45	35	25	24	20	**27**	20	**Decrease**	<0.001 *
PBIs	45	55	45	35	33	41	**47**	38	**Decrease**	<0.001 *
AABPIs	33	52	43	32	31	32	**39**	26	**Decrease**	<0.001 *
Fluoroquinolones	46	53	41	31	29	26	**30**	25	**Decrease**	<0.001 *
Sulfonamides	26	39	33	25	24	18	**25**	18	**Decrease**	<0.001 *

PBIs: penicillins and beta-lactamase inhibitors; AAPBIs: anti-pseudomonal activity penicillins and beta-lactamase inhibitors. Trend changed in bold. * Cochran Armitage test; *p*-value significant.

**Table 4:** Cross-correlation analysis of antimicrobial resistance and antimicrobial consumption.

Antimicrobial Resistance	Significant Correlation with Antimicrobial Consumption	Lag Time (Month)	Coefficient of Correlation (r)
Aminoglycosides	Aminoglycosides	3	0.44
Cephalosporins	Fourth-generation cephalosporin	3	−0.48
		4	−0.45
		5	−0.44
	Cephalosporins	1	0.43
	Fluoroquinolones	1	0.38
		2	0.45
		3	0.35
Anti-pseudomonal activity penicillin and beta-lactamase inhibitors	Fourth-generation cephalosporin	1	−0.47
		2	−0.49
		3	−0.43
	Cephalosporins	1	0.46
	Fluoroquinolones	1	0.47
		2	0.40
		3	0.33
Penicillin and beta-lactamase inhibitors	Penicillin and beta-lactamase inhibitor	0	0.43
		1	0.52
		2	0.52
	Cephalosporins	1	0.50
	Fluoroquinolones	1	0.57
Fluoroquinolones	Cephalosporins	1	0.43

Aminoglycosides: amikacin, gentamicin, tobramycin; Fourth-generation cephalosporin: cefepime; Cephalosporins: ceftriaxone, cefotaxime, ceftazidime; Penicillin and beta-lactamase inhibitors: amoxicillin-clavulanic acid, ampicillin-sulbactam; Anti-pseudomonal activity penicillin and beta-lactamase inhibitors: piperacillin-tazobactam, ticarcillin-clavulanic acid; Fluoroquinolones: ofloxacin, norfloxacin, ciprofloxacin, levofloxacin, moxifloxacin.

**Table 5:** Parameters of multivariate transfer function (dynamic regression) models.

Models	Lag-Time (Month)	Adjustment	Parameters (SE)	AIC	R^2^
Aminoglycoside resistance					
Moving average	1	-	−0.65 (0.20)	101.5	58%
Aminoglycosides	1	-	0.98 (0.04)		
Cephalosporin resistance					
Cephalosporins	1	Adjustment in 2 principal components	−4.6 (1.2)	145.5	47%
Fourth-generation cephalosporin	3 to 5				
Fluoroquinolones	1 to 3		−0.95 (1.4)		
AAPBI resistance					
Cephalosporins	1	Adjustment in 2 principal components	6.47 (1.54)	165.5	48%
Fourth-generation cephalosporin	1 to 3		2.15 (1.94)		
Fluoroquinolones	1 to 3				
PBI resistance					
Cephalosporins	1	Adjustment in 2 principal components	5.6 (1.1)	169.2	55%
PBIs	0 to 2				
Fluoroquinolones	1		−0.16 (1.48)		
Fluoroquinolone resistance					
Cephalosporins	1	Adjustment in 1 principal component	4.6 (2.13)	175.2	46%
Fluoroquinolones	0 to 2				
Moving average	1		0.65 (0.14)		

Parameters should be interpreted as a weighting of their involvement in the model; SE: standard error; AIC: akaike information criterion; R^2^: determination coefficient; Aminoglycosides: amikacin, gentamicin, tobramycin; Fourth-generation cephalosporin: cefepime, Cephalosporins: ceftriaxone, cefotaxime, ceftazidime; Penicillin and beta-lactamase inhibitors (PBIs): amoxicillin-clavulanic acid, ampicillin-sulbactam; Anti-pseudomonal activity penicillin and beta-lactamase inhibitors (AAPBIs): piperacillin-tazobactam, ticarcillin-clavulanic acid; Fluoroquinolones: ofloxacin, norfloxacin, ciprofloxacin, levofloxacin, moxifloxacin.

## Data Availability

The raw data supporting the conclusions of this article will be made available by the authors on request.

## Abbreviations

The following abbreviations are used in this manuscript:

AAPBI	Anti-pseudomonal activity penicillin and beta-lactamase inhibitors
AMC	Antimicrobial consumption
AMR	Antimicrobial resistance
ARIMA	Autoregressive integrated moving average
ARIMAX	Autoregressive integrated moving average with explanatory variables
AST	Antimicrobial susceptibility testing
COVID-19	Coronavirus disease 2019
DDD	Defined daily doses
DR	Dynamic regression
DRPC	Dynamic regression by principal component
LTF	Linear transfer function
PBI	Penicillin and beta-lactamase inhibitor
SARS-CoV-2	Severe acute respiratory syndrome coronavirus 2
WHO	World Health Organization
